# Low TYROBP expression predicts poor prognosis in multiple myeloma

**DOI:** 10.1186/s12935-024-03304-6

**Published:** 2024-03-28

**Authors:** Hong Luo, Chengyun Pan, Li Wang, Lin Zheng, Shuyun Cao, Xiuying Hu, Tianzhen Hu, Naiqin Zhao, Qin Shang, Jishi Wang

**Affiliations:** 1 Department of Hematology, Affiliated Hospital of Guizhou Medical University, Guiyang, 550004 China; 2 Department of Clinical Medical School, Guizhou Medical University, Guiyang, 550004 China; 3 National Clinical Research Center for Hematologic Diseases, The First Affiliated Hospital of Soochow University, Jiangsu, 215006 China

**Keywords:** Biomarker, TYROBP, Multiple myeloma, Prognosis, Adhesion, Migration

## Abstract

**Background:**

Multiple myeloma (MM) is the second most common refractory hematologic cancer. Searching for new targets and prognostic markers for MM is significant.

**Methods:**

GSE39754, GSE6477 and GSE24080 were downloaded from the Gene Expression Omnibus (GEO) database. Differentially expressed genes (DEGs) in MM versus healthy people from GSE39754 and GSE6477 were screened using limma package, and MM-related module genes were chosen with the use of Weighted gene co-expression network analysis (WGCNA), and the two were intersected using ggVennDiagram for obtaining MM-related DEGs. Gene Ontology (GO) and Kyoto Encyclopedia of Genes and Genomes (KEGG) analyses were carried out. Then, protein–protein interactions (PPI) analysis in String database was used to obtain hub genes, while prognosis was analyzed by survival package in GSE24080. Receiver operating characteristic (ROC) curve was adopted for evaluating diagnostic value of hub genes. Besides, univariable/multivariable Cox regression were employed to screen independent prognostic biomarkers. Gene set enrichment analysis (GSEA) was used to find possible mechanism. Finally, western-blotting and reverse transcription-polymerase chain reaction (RT-PCR) verify TYROBP expression within MM and healthy people. We performed cell adhesion and transwell assays for investigating TYROBP function in MM cell adhesion and migration.

**Results:**

Through differential analyses, 92 MM-related DEGs were obtained. 10 hub genes were identified by PPI and CytoHubba. Their diagnostic and prognostic significance was analyzed. Down-regulation of genes like TYROBP, ELANE, MNDA, and MPO related to dismal MM prognosis. Upon univariable/multivariable Cox regression, TYROBP independently predicted MM prognosis. GSEA pathway was enriched, indicating that TYROBP expression affected MM development via cell adhesion molecular pathway. Upon Western-blotting and RT-PCR assays, TYROBP expression among MM patients decreased relative to healthy donors. Cell adhesion and transwell migration assays revealed increased MM cell adhesion and decreased migration upon TYROBP up-regulation.

**Conclusion:**

In summary, TYROBP is a potential prognostic marker for MM.

**Supplementary Information:**

The online version contains supplementary material available at 10.1186/s12935-024-03304-6.

## Introduction

Multiple myeloma (MM), a refractory disease, shows the typical feature of abnormal clonal plasma cell proliferation, and it occupies approximately 10% of hematological malignancies [1]. Malignant proliferation of plasma cells may lead to osteolytic osteopathy, kidney damage, anemia and hypercalcemia [2]. Currently, the diagnostic and prognostic biomarkers for MM have been identified, but MM still has high morbidity and mortality rates [3], remains incurable, and exhibit poor prognosis for patients [4]. As a result, it is of great significance to search for effective prognostic markers and explore new targets for the clinical improvement of MM prognosis.

Tyrosine kinase binding protein TYROBP(DAP12) is a transmembrane signal transduction polypeptide, which binds to a variety of receptors activated on the surface of immune cells and regulates immune cell function through signal transduction [5, 6]. TYROBP regulates the production of inflammatory factors in immune cells, thus mediating the inflammatory response in the body [7]. Inflammation is not only closely related to immune response and leads to the occurrence of numerous diseases, but is also an important factor for cancer genesis and progression [8, 9]. When TYROBP binds to its receptor TREM-1, it initiates the intracellular signaling cascade by means of synergistic action with TLR signaling, thus amplifying the inflammatory response in vivo [10]. In addition, the TYROBP-SYK pathway promotes TGF-β secretion in macrophages, while this in term promotes tumor progression [11]. The TYROBP/ITAM2 signaling pathway can promote the malignant growth of liver cancer cells [12]. Moreover, studies have reported that TYROBP is a potential pathogenic oncogene in gastric cancer, renal clear cell carcinoma, and glioma [13–15]. TYROBP is extensively investigated as the possible target and prognostic marker for the development and metastasis of renal cancer [16], osteosarcoma [17, 18] and breast cancer [19]. In conclusion, TYROBP is tightly associated with cancer progression. However, its function in MM remains unclear.

In this study, DEGs associated with MM were screened based on GEO database. Finally, TYROBP was found to be an independent prognostic factor for MM. Compared with healthy controls, TYROBP expression was significantly downregulated in MM patients. In accordance with GSEA results of low- and high-TYROBP-expression groups, TYROBP might probably impact MM cell migration and adhesion through influencing changes in cell adhesion molecules, leading to poor prognosis of MM patients. Based on bioinformatics analysis and experimental verification, this study revealed the potential role and prognostic value of TYROBP in MM. Collectively, findings in this study contribute to better understanding the potential molecular mechanism underlying MM genesis and progression.

## Materials and methods

### Microarray data

Gene expression datasets GSE39754, GSE6477 and GSE24080 were obtained based on NCBI-GEO (http://www.ncbi.nlm.nih.gov/geo). GSE39754 data were acquired with GPL5175 platform ([HuEx-1_0-st] Affymetrix Human Exon 1.0 ST Array [transcript (gene) version]), which involved purified plasma cell samples obtained in 170 MM cases together with 6 normal subjects. GSE6477 data were obtained from the GPL96 platform ([HG-U133a] Affymetrix Human Genome U133A Array), which included purified plasma cell samples collected in 103 cases as well as 15 normal subjects. GSE24080 data were gathered from the GPL570 ([HG-U133_Plus_2] Affymetrix Human Genome U133 Plus 2.0 Array), which contained bone marrow plasma cell gene expression profiles for survival analysis and GSEA.

### DEGs identification and weighted gene co-expression network analysis (WGCNA)

The DEGs (DEG1 and DEG2) in MM patients and normal donor samples from GSE39754 and GSE6477 datasets were explored using limma package, with the threshold being set to an adjusted P < 0.05 and |log2FC| ＞ 1. Visualization analysis was performed by plotting heat maps and volcano maps using the circlize package and ggplot2, respectively. In addition, we utilized R-package software WGCNA for constructing and analyzing co-expression network of GSE39754 dataset. The modules with the highest correlation with MM traits were obtained, meanwhile, the module genes were acquired.

### DEGs related to MM and functional enrichment analysis

In this work, we drew Venn diagram for obtaining MM-related DEGs. The biological functions of DEGs related to MM were assessed using GOplot and ccgraph for Gene Ontology (GO) and Kyoto Encyclopedia of Genes and Genomes (KEGG) pathway enrichment, respectively. Notably, the biological functions were divided into biological process (BP), molecular function (MF), and cellular component (CC) categories.

### Establishment of PPI network and identification of hub gene

Online String database (http://string-db.org/cgi/input.pl) was adopted for predicting protein interaction. In brief, MM-related DEGs were uploaded into the String database and the smallest interaction score of 0.4 was selected to obtain the result file. Meanwhile, the result was analyzed visually using Cytoscape, and the top 10 MCC scoring genes were determined by the cytoHubba plug-in, which were denoted as hub genes.

### Receiver operating characteristic (ROC) curve and Kaplan–Meier (KM) survival curve

In GSE39754 and GSE6477 dataset, diagnostic curves of a single hub gene were drawn using "pROC" to evaluate the sensitivity and specificity of a single hub gene in the diagnosis of MM. In the GSE24080 dataset, the survival kit was utilized for analyzing the survival of the single hub gene with high or low expression and the prognostic significance of high- or low-TYROBP-expression group under different clinical characteristics. In line with the median value of single gene expression, MM patients were divided into high- and low- expression groups.

### GSEA functional enrichment analysis

In the GSE24080 dataset, all genes in high- or low-TYROBP-expression group were analyzed by using package R clusterProfiler and package org.Hs.eg.db (version 3.15.0), respectively, with thresholds being set as |NES|> 1 and P < 0.05.

### Patient samples collection and ethics consent

In this study, newly diagnosed samples from MM patients at Department of Hematology, Affiliated Hospital of Guizhou Medical University from 2021 to 2023 were selected by random sampling. All these patients satisfied the diagnostic criteria for MM recommended by National comprehensive cancer network (NCCN)2022. In addition, 22 healthy donors and 35 patients were collected. Mononuclear cells were extracted from all the collected samples. Based on the Helsinki Declaration, the informed consent was first obtained in writing. We have obtained the approval of the institutional ethics committee and the right of informed consent from the patients in advance. Additional file 1: Table S1 provides characteristics of multiple myeloma patients and healthy donors.

### Cell lines and cell transfection

Human myeloma cell lines U266 and RPMI8226 were obtained from the Shanghai FuHeng Biology in 2022. All cell lines were authenticated by short tandem repeat profiling. All cells were cultured in RPMI-1640 containing 10% Fetal bovine serum (FBS) and 1% Penicillin–Streptomycin. The cells were incubated at 37 °C in an atmosphere of 5% CO2.

We obtained human TYROBP overexpression cloned lentiviral particle (L-TYROBP) in Genechem Co., Ltd. (Shanghai, China) for TYROBP transfection in line with specific protocols. Empty vector (EV)-transfected cells (U266 and RPMI8226) were adopted to be controls. Following amplification, cells were maintained within RPMI-1640 medium that contained 10%FBS for a 5-day period. Then, U266 and RPMI8226 cells stably transfected with L-TYROBP were screened with puromycin(1 ug/ml), and Western-Blotting and RT-PCR were performed to verify the transfection efficiency.

### Western-Blotting assay

Radio immunoprecipitation assay (RIPA) lysate that contained 1% Phenylmethylsulfonyl fluoride (PMSF) was used to lyse MM cells (cell lines and clinical samples), while BCA kit was utilized to detect protein content. Afterwards, 30 ug protein sample was loaded onto the 12% SDS-PAGE gel and the isolated proteins were transferred onto PolyVinylidene Fluoride (PVDF) membranes. Thereafter, 5% defatted milk was added to seal PVDF membranes for at least 2 h, and Twen-containing triple buffered brine was added to wash membranes. Later, membranes were subjected to overnight incubation using target antibody, washing again, and 45-min incubation using secondary antibody. After washing again, target protein levels were measured through the electrochemiluminescence method, while imageJ_v1.8.0 was utilized to detect gray value.

### RT-PCR

In line with specific protocols, the Trizol reagent was utilized to extract total RNA, which was later prepared in cDNA through reverse transcription using a reverse transcription kit. Afterwards, SYBR Green kit (Tiangen Biotechnology) and RT-PCR primer (Generay Biotech) were utilized to test cDNA in the sample. The Bio-Rad instrument was used to test the sample cyclic threshold (CT) value. With beta-actin being a reference, relative target gene level was calculated through comparing the CT value (2^ − ΔCT^). Primers below were utilized in the present work:

β-actin F, 5ʹ-CTACCTCATGAAGATCCTCACCGA-3ʹ;

β-actin R, 5ʹ-TTCTCCTTAATGTCACGCACGATT-3ʹ;

TYROBP F, 5ʹ-TCCTGCTGGCTGTAAGTGA-3ʹ;

TYROBP R, 5ʹ-CATCCGACCTCTGACCCT-3ʹ.

### Cell adhesion

Cell adhesion was analyzed using the cell adhesion detection kit (Bestbio, BB-48120). Briefly, 100 ul coating solution was added into each well of the 96-well plate, and the 96-well plate was later placed in a 4 ℃ refrigerator overnight. After removing the coating liquid, the 96-well plate was washed with 100ul washing liquid for 1–3 times after it was completely dried. Subsequently, cells (5 × 10^4^ /100 ul/well) were incubated in an incubator under 37 °C for 2 h, and 5 replicate wells together with a control group were set. Later, the culture plate was removed, the medium was discarded, and relevant medium was added to wash the plate for 2–3 times, followed by the addition of 100 ul freshly prepared medium into every well. Afterwards, 10ul cell staining solution was introduced to incubate at 37 ℃ for 0.5–3 h. The absorbance at 450 nm was detected with an enzymoleter.

### Transwell migration assay

In the transwell migration assay, serum-free medium was added to re-suspend the cell suspensions to 10^5^ cells/ml. Thereafter, 100 ul cell suspensions were introduced into top chamber of the 24-well plate transwell chamber, while 600 ul of 20% FBS was introduced into lower chamber for 24 h incubation at 37 ℃ and 5% carbon dioxide conditions. After 24 h, the cell medium was abandoned, cells were subjected to 30-min fixation using paraformaldehyde and 30-min staining using 0.1% crystal violet. Those stained cells were then cleaned thrice using PBS and observed under the inverted microscope.

### Data analysis

The wilcox.test test was conducted for comparing gene levels between patient and healthy samples in the dataset. Prognostic factors for MM were identified through univariable and multivariable Cox regression. Comparisons of the two groups were conducted with Student’s *t*-test. All experiments were conducted at least three times. A mean ± SD was used for all quantitative data. *P *< 0.05 was determined as statistically significant.

## Results

### Identification of DEGs and susceptibility modules

**Fig. 1:**
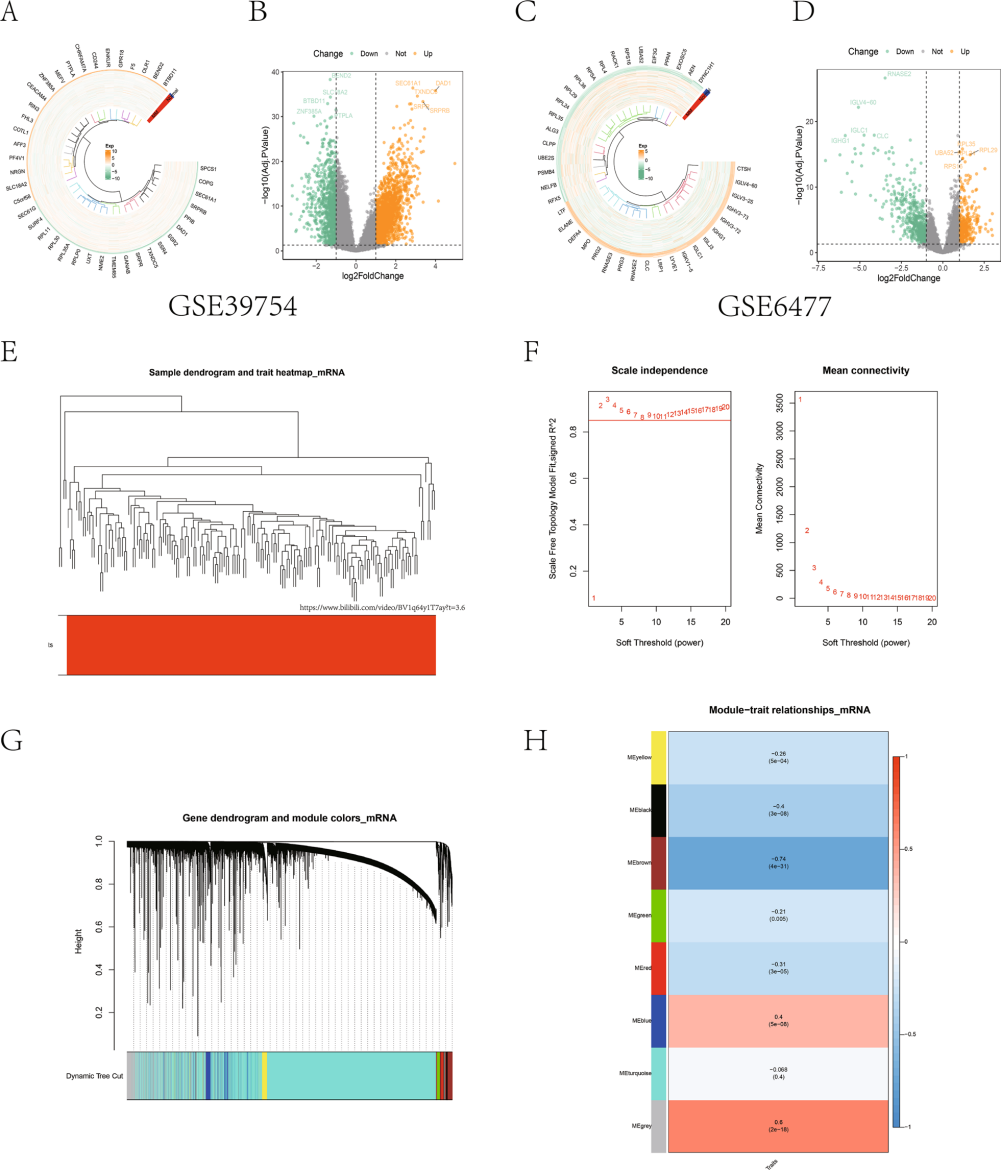
DEGs and WGCNA. Heatmaps and volcano plots showing DEGs in GSE39754 (**A**, **B**) and GSE6477 (**C**, **D**). **E** Sample clustering conducted according to GSE39754 dataset. **F** Scale-free fit index and the mean connectivity of 1–20 soft-threshold power. **G** Gene tree diagram. The colored line underneath the tree diagram indicates modules examined with dynamic tree cutting. **H** Heatmap showing relation of feature genes in the module with multiple myeloma

DEGs between MM patients and normal donors from the GEO datasets (GSE39754 and GSE6477) were screened. As a result, DEG1, including 1022 down-regulated genes and 1996 up-regulated genes, were screened from GSE39754 dataset; whereas DEG2 (including 548 down-regulated genes while 356 up-regulated ones) were obtained from GSE6477 dataset, as displayed in the forms of heat maps and volcano maps (Fig. 1A–D). Later, WGCNA was performed on GSE39754 dataset. Firstly, cluster analysis was conducted to observe whether there were outliers (two samples were removed, while 174 were retained), as shown in Fig. 1E. Secondly, according to the gene clustering of all samples, a suitable soft threshold was selected for network topology analysis. Typically, the value was selected in the sense of R^2^ ≥ 0.85, and finally 8 was chosen to be the soft threshold (Fig. 1F). Then, the topological matrix obtained by the differences between genes was used for clustering. The adjacency and divergence coefficient between genes were calculated, and the tree was divided into different modules (with at least 50 genes in each module) by the dynamic clipping technique, as shown in Fig. 1G. Eventually, the heat map showing relationship of modules was drawn, then module gene most significantly correlated with tumor traits was screened. According to our results, the gene module most closely correlated with phenotype was brown module (601 genes, Fig. 1H), which was selected for later analyses.

### Functional annotation of MM-related DEGs and identification of hub genes

**Fig. 2:**
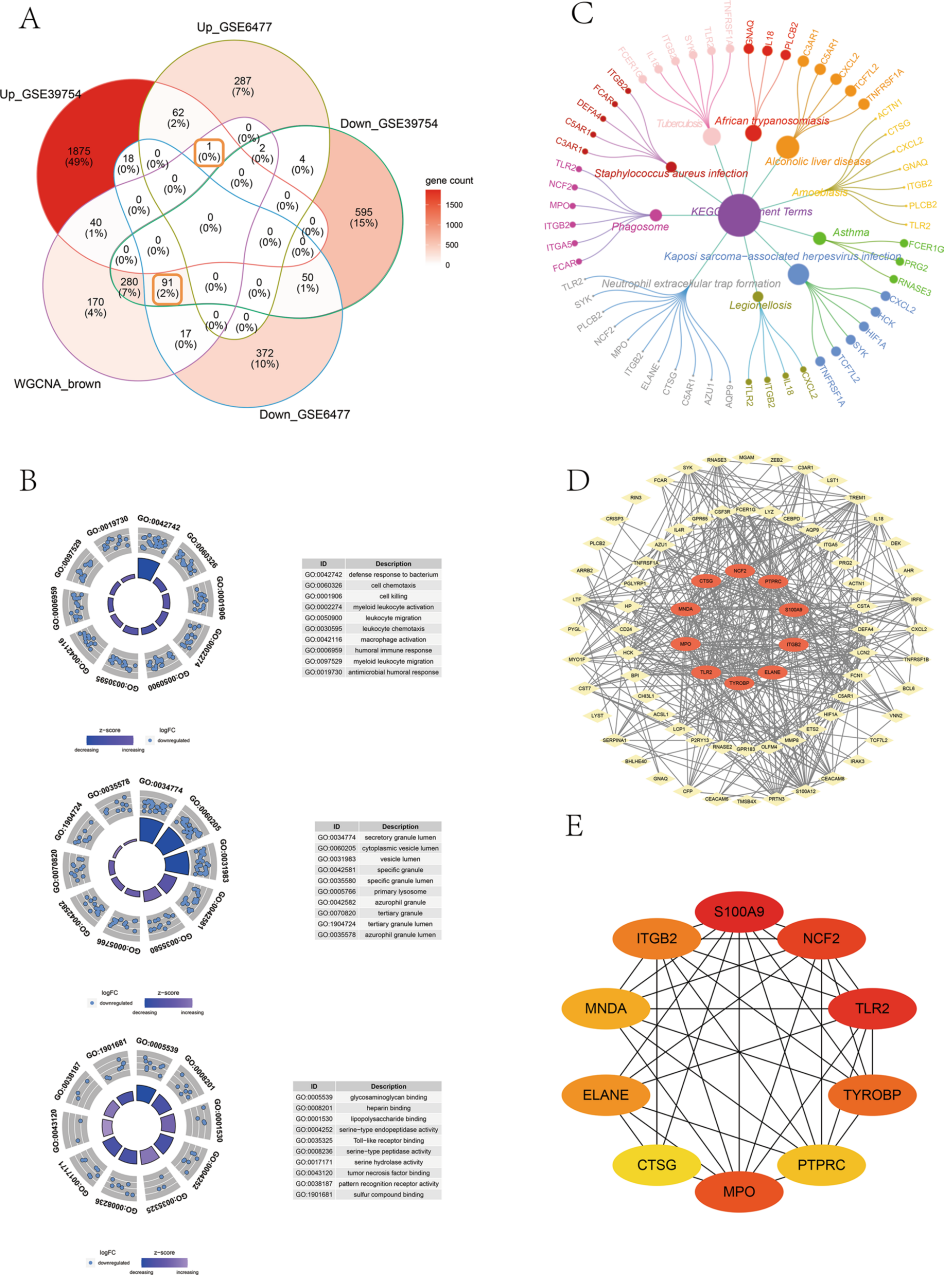
Enrichment analysis and hub gene identification. **A** Venn diagram showing the number of DEGs related to multiple myeloma. **B** GO functional annotation of DEGs associated with multiple myeloma. **C** KEGG analysis on DEGs associated with multiple myeloma. **D**, **E** PPI network analysis showing the hub genes

Firstly, DEG1 and DEG2 were intersected with Brown module genes to obtain altogether 204 DEGs, including 63 with up-regulation whereas 141 with down-regulation, and 92 of them were MM-related DEGs (Fig. 2A). Secondly, the MM-related DEGs were exposed to GO and KEGG analyses. In total, 680 GO items were mainly enriched and analyzed, and it was found that the BP terms enriched were mainly in defense response to bacterium and cell ﻿chemotaxis, the CC terms enriched included secretory granule lumen and cytoplasmic vesicle lumen, and the MF terms enriched were glycosaminoglycan binding, heparin binding and lipopolysaccharide binding (Fig. 2B). Furthermore, KEGG enrichment analysis revealed some signaling pathways involved in MM-related DEGs (Fig. 2C), mainly including Neutrophil extracellular trap formation, Amoebiasis and Phagosome pathways. Moreover, a PPI network that involved 78 proteins with 78 nodes and 434 edges was established by Cytoscape on the basis of String database (Fig. 2D). We chose the top 10 genes in the MCC score to be hub genes for further analysis (Fig. 2E), including S100A9, NCF2, TLR2, TYROBP, PTPRC, MPO, CTSG, ELANE, MNDA and ITGB2.

### Diagnostic significance and survival analyses of hub genes

**Fig. 3:**
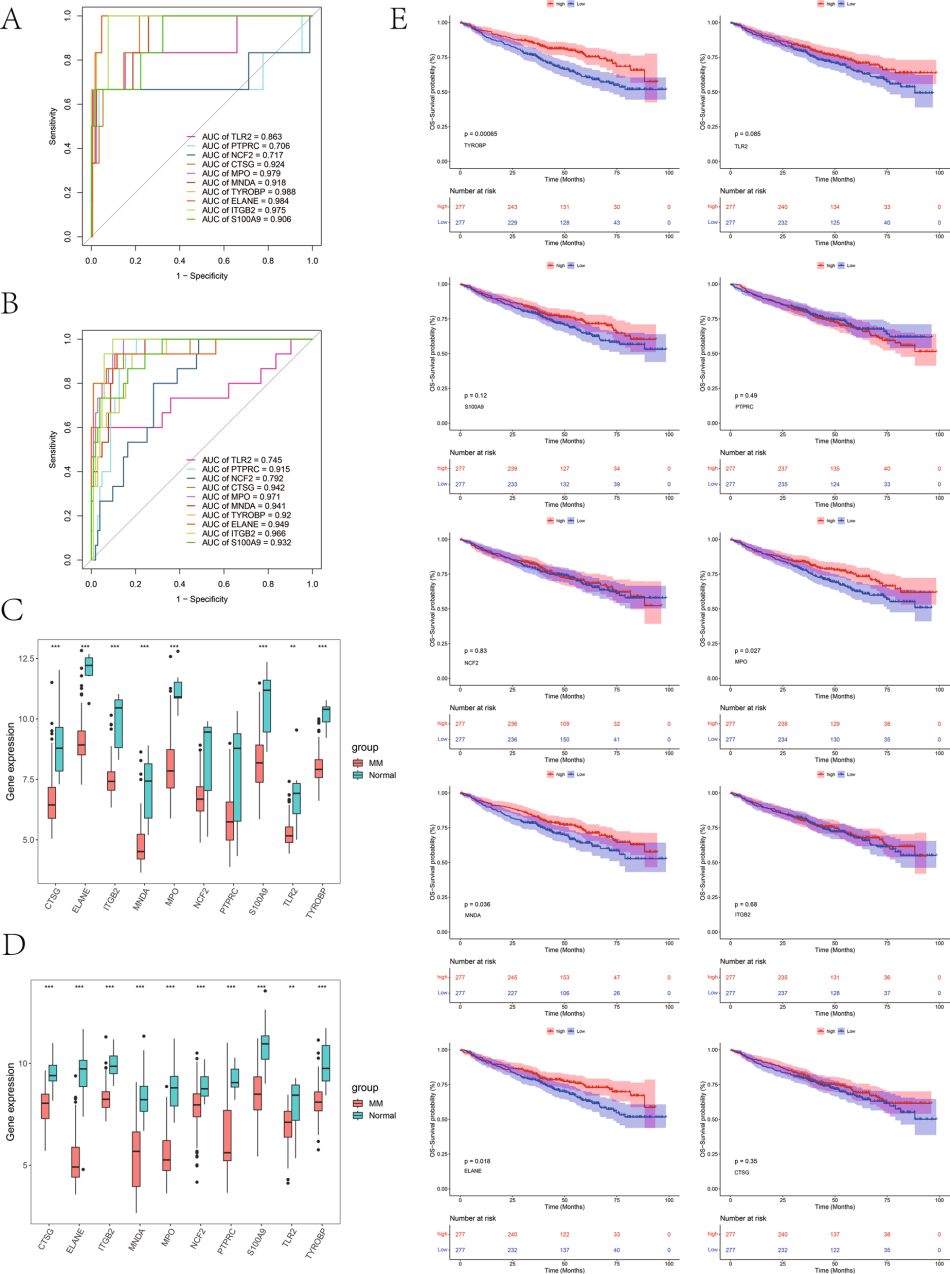
Diagnosis and survival analyses on hub genes. **A**, **B** ROC analysis of hub genes diagnostic value in multiple myeloma based on GSE39754 and GSE6477. **C**, **D** Expression of hub genes based on GSE39754 and GSE6477. **E** Survival analysis on hub genes

First, in the GSE39754 and GSE6477 datasets, the ROC diagnostic curve of a single hub gene was plotted. As observed from Fig. 3A, there were 8 genes with AUC values greater than 0.8 in the GE39754 dataset (TLR2, CTSG, MPO, MNDA, TYROBP, ELANE, ITGB2, and S100A9). According to Fig. 3B, there were 8 genes (PTPRC, CTSG, MPO, MNDA, TYROBP, ELANE, ITGB2, and S100A9) with AUC values greater than 0.8 in the GSE6477 dataset. Then, the differential analysis of hub gene between MM and health donors was verified in GSE39754 and GSE6477 datasets (Fig. 3C, D), as a result, hub gene expression decreased in both datasets. Finally, based on median expression of single gene, we classified hub genes as high- or low-expression group. For GSE24080 dataset, survival analysis was conducted for hub genes in high- or low-expression group. The results suggested that only TYROBP, ELANE, MNDA and MPO genes showed significant differential expression in high- versus low-expression groups (Fig. 3E).

### Independent prognostic significance of hub genes and GSEA

**Fig. 4:**
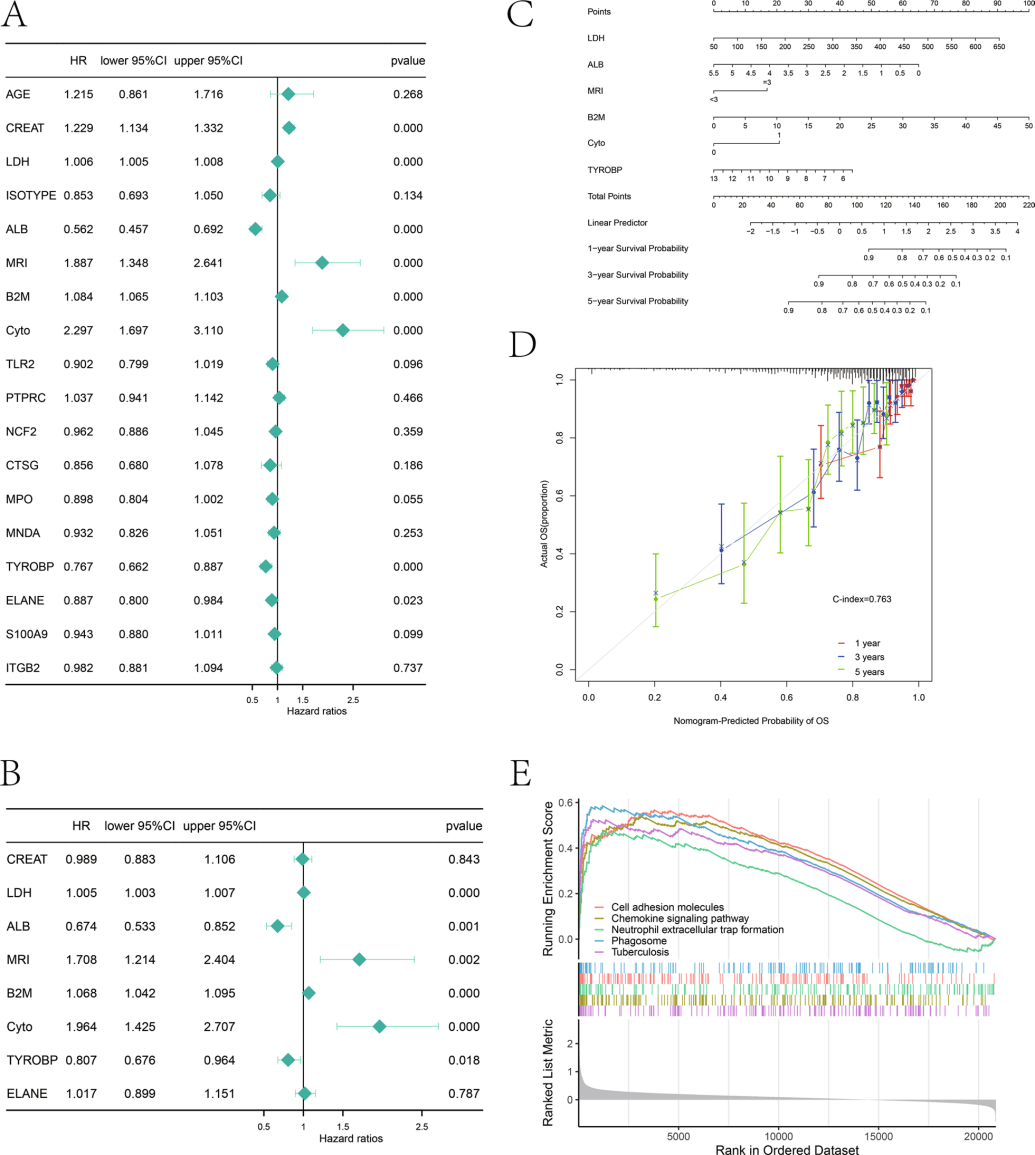
Relationship between hub genes and multiple myeloma prognosis as well as GSEA enrichment analysis. **A** Forest map according to univariate Cox regression of OS for multiple myeloma patients. **B** Forest map according to multivariate Cox regression of OS in multiple myeloma patients. **C** Nomogram predicting the survival rate. **D** Calibration curve predicting the performance of nomogram model. **E** GESA enrichment analysis exploring the potential functions

We utilized Cox regression model for analyzing relation of AGE, CREAT, LDH, ISOTYPE, ALB, MRI, B2M, Cyto and Hub genes levels and survival time among MM patients. As revealed by univariable and multivariable regression, LDH, ALB, MRI, B2M, Cyto and TYROBP were used as the independent prognostic factors for MM (Fig. 4A, B). Later, we built a nomogram by incorporating the independent prognostic factors for predicting 1-, 3- and 5-year survival rates in MM patients (Fig. 4C). Moreover, the corresponding calibration curve was drawn, and its C-index was 0.763, suggesting the superior performance of this nomogram (Fig. 4D). To further explore the possible molecular function of TYROBP gene within multiple myeloma, we carried out GSEA on genes of high- and low-TYROBP-expression groups. KEGG analysis revealed significant changes in cell adhesion molecules, chemokine pathways, neutrophil peripheral trap formation, phagocytic vesicles, and Tuberculosis signaling pathways in high- and low-TYROBP-expression groups (Fig. 4E). It was hypothesized that TYROBP expression might contribute to the adhesion and migration of MM by influencing the changes of cell adhesion molecules.

### Survival analysis of high- and low-TYROBP-expression groups under different clinical characteristics

**Fig. 5:**
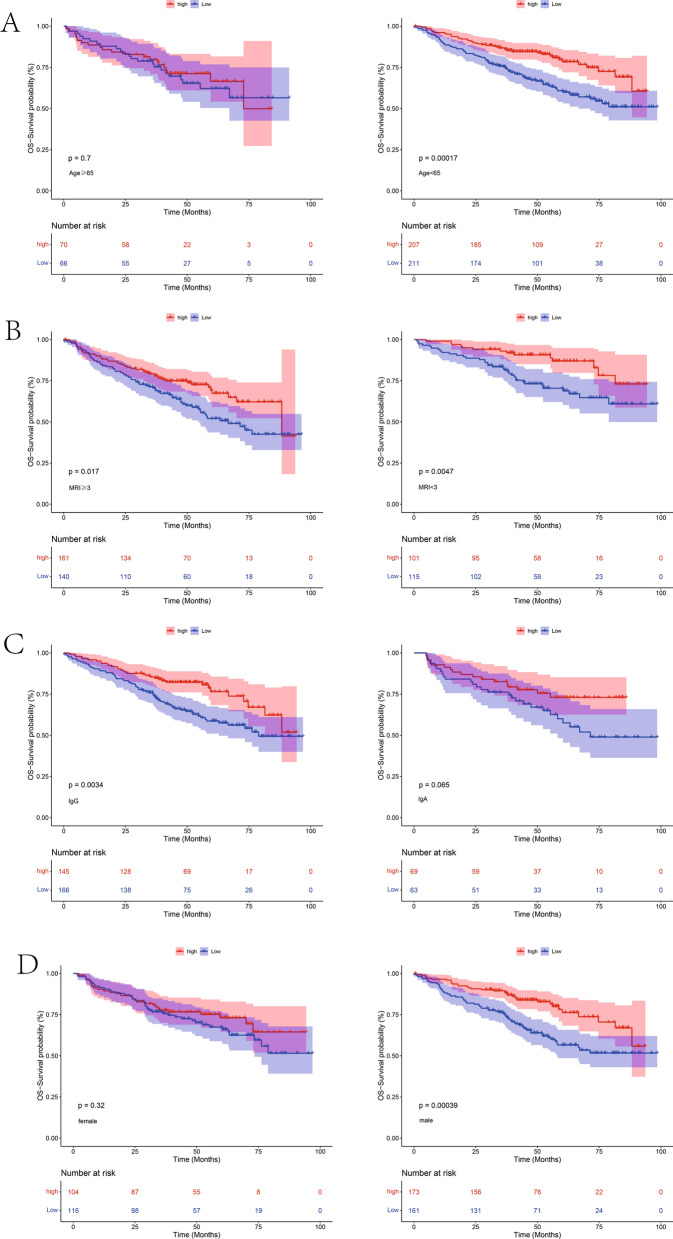
Kaplan–Meier survival curve predicts the survival probability under different clinical features

MM patients were grouped according to different clinical features, such as AGE, MRI, ISOTYPE, and SEX. Thereafter, the classification groups with different clinical features were further grouped according to the high- and low-TYROBP-expression groups for KM analysis. As a result, there was prognostic significance when the patients younger than 65 years old, having MRI lesions, IGA type, and female patients were grouped. This finding indicated the survival differences between the high- and low-TYROBP-expression groups for most clinical features (Fig. 5A–D).

### TYROBP was down-regulated in MM and TYROBP up-regulation promoted the adhesion of MM and decreased its migration

**Fig. 6:**
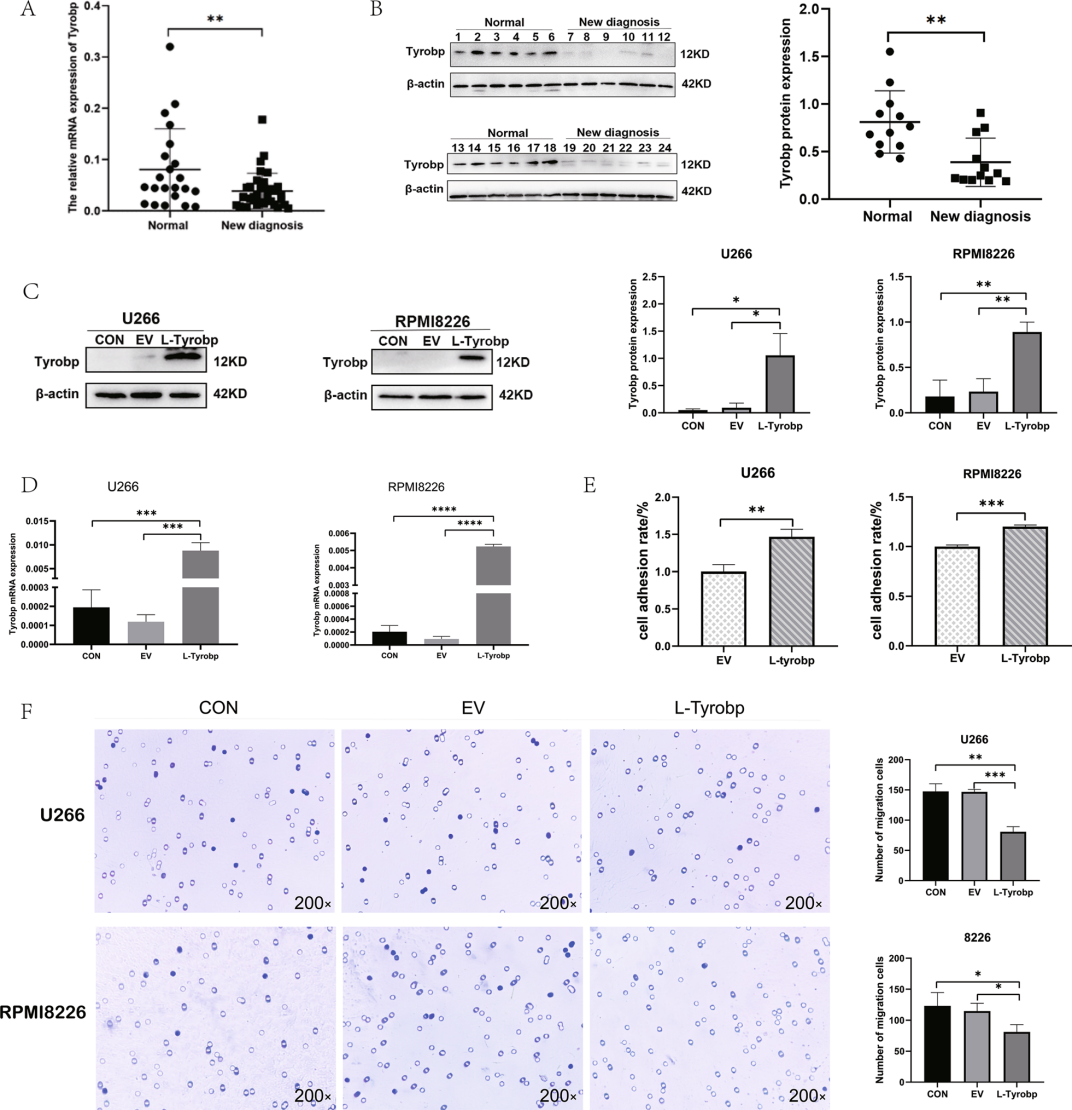
TYROBP is lowly expressed in multiple myeloma and overexpression of TYROBP promotes myeloma cells adhesion and inhibits their migration. **A** RT-PCR conducted to analyze the mRNA expression of TYROBP. **B** Western blot was employed to determine the expression level of TYROBP in multiple myeloma. **C** TYROBP overexpression determined by Western blot in U266 and RPMI8226 cells. **D** RT-PCR revealing the successful up-regulation of TYROBP. **E** Adhesion assay suggesting that TYROBP facilitated the adhesion of U266 and RPMI8226 cells. **F** Transwell migration assay proving that TYROBP overexpression inhabited the migration of U266 and RPMI8226 cells. *p < 0.05, **p < 0.01 and ***p < 0.001

Next, TYROBP expression in MM and its prognostic value were verified by using clinical samples and conducting in vitro cell experiments. To verify TYROBP levels among MM patients, we examined TYROBP mRNA and protein expression within bone marrow blood samples from MM patients and normal donors. According to RT-PCR analysis, TYROBP mRNA expression among MM patients markedly decreased relative to normal donors (Fig. 6A). Based on Western-Blotting analysis, TYROBP protein expression among MM patients significantly decreased relative to normal donors (Fig. 6B). It is well known that changes in cell adhesion molecules are important factors for tumor metastasis. Therefore, we investigated how TYROBP affected myeloma cell adhesion and migration when TYROBP was highly expressed in MM cells. As revealed by Western-blotting assays and PCR, TYROBP expression successfully increased in myeloma cells (Fig. 6C, D). Cell adhesion experiments suggested that TYROBP up-regulation promoted cell adhesion (Fig. 6E). Transwell migration assay demonstrated that cell migration ability was weakened when TYROBP was highly expressed (Fig. 6F).

## Discussion

MM tends to occur in the elderly, with a median age of diagnosis being 69 years [20]. Its incidence may probably increase year by year with the progression of the aging society and the development of disease understanding and diagnosis and treatment technologies. Despite great improvements in existing treatments, the overall prognosis of MM patients remains poor, and MM is still a refractory disease due to relapse and drug resistance [21]. Consequently, the search for new prognostic biomarkers for MM patients provides new ideas for diagnosing and treating myeloma and improving patient outcomes.

Bioinformatics analysis was conducted in the present work to integrate two datasets, GSE39754 and GSE6477, for the sake of identifying 92 DEGs most significantly related to MM through a series of analyses. Further GO and KEGG pathway analyses indicated that these 92 DEGs were significantly enriched in cell chemotaxis, glycosaminoglycan binding, lipopolysaccharide binding, and Neutrophil extracellular trap formation. Cell chemotaxis is an important factor for promoting tumor progression and metastasis [22]. Meanwhile, glycosaminoglycan is an important component of tumor microenvironment, which can affect tumor growth, migration, invasion and angiogenesis through various ways such as binding to cytokines, thus affecting tumor progression. It has great potential in the targeted therapy of cancer [23–25]. Lipopolysaccharide (LPS) binds to the TLR4 on tumor cell surface for activating NF-KB pathway to regulate tumor cell invasion and migration [26]. Neutrophil extracellular trap formation has a critical effect on tumor genesis and progression, promoting tumor angiogenesis, metastasis and diffusion [27]. These results suggest that the MM-related DEGs may influence MM biological behavior through these pathways, thereby regulating MM genesis and progression. For identifying genes related to MM development, the PPI network was constructed to screen key hub genes, among which, the 10 most significant ones were recorded as hub genes. Then, these hub genes were subjected to ROC diagnosis and survival analyses, and univariable and multivariable regression was performed on hub genes and clinical characteristics. As a result, LDH, ALB, MRI, B2M, Cyto and TYROBP were identified as independent prognostic factors.

TYROBP represents the type I transmembrane adaptor protein whose cytoplasmic domain contains an immune receptor tyrosine activation motif ITAM. After TYROBP combines with the activated receptor, SRC kinase can phosphorylate ITAM motifs to its two conserved tyrosine residues, thereby recruiting and activating intracellular protein kinases including SYK, which affects multiple downstream effector molecules. Phosphatidylinositol 3-kinase (PI3K), phospholipase Cγ (PLCγ) and small GTPase RAS can be mobilized to affect cell transcriptional activation, proliferation and survival, cytokine secretion, and phagocytosis [6]. Notably, the aberrant TYROBP expression is related to the genesis and progression of various disorders, and it is related to the pathogenic mechanism of Alzheimer's disease [28] and cognitive dysfunction. TYROBP on lung macrophages can mediate acute non-infectious lung tissue injury through affecting transendothelial migration of neutrophils [29]. TYROBP promotes the development of inflammation-mediated atrial fibrillation through PI3K-AKT pathway [30]. As discovered from an article on neuropathic pain, TYROBP promoted inflammatory factor levels like TNF-α, IL-6, and inflammation-related genes, showing a pro-inflammatory response [31]. When TYROBP receptors such as TREM2 bind to ligands to form the ligand-receptor complexes, ITAM phosphorylation in the cytoplasmic region of TYROBP is triggered to recruit and mediate SYK phosphorylation, thereby activating the downstream signaling pathways like PI3K and MAPK pathways [32]. PI3K exerts an important effect on a variety of cell activities, and shows abnormal activation in cancer, thereby participating in tumor genesis and development. Inhibitors targeting this signaling pathway, some of which are approved for treating cancer in clinic, have certain significance for increasing cancer patients survival rates [33]. MAPK pathway is related to regulating cell growth, cycle, differentiation, development, apoptosis, and other important physiological processes. Human cancer is tightly associated with disorder of this pathway, which can promote tumor proliferation, survival, invasion, metastasis, extracellular matrix degradation and angiogenesis [34]. As mentioned in the preface, a number of studies have also proved that TYROBP is a prognostic marker of tumors and affects tumor progression. In our study, we found that cell adhesion molecules, chemokine signaling pathway, and neutrophil extracellular trap were mainly enriched by GSEA enrichment analysis on high- and low-TYROBP-expression groups, which were closely associated with tumor migration and invasion. Cell adhesion related molecules are important factors for determining tumor migration and invasion, which also affect the progression of MM [35]. To further analyze how TYROBP affected MM progression, we verified the low expression of TYROBP in multiple myeloma bone marrow samples and in vitro cell adhesion assay found that the high expression of TYROBP promoted cell adhesion. Transwell migration assay suggested that the migration of myeloma cells was reduced when TYROBP was highly expressed. Based on the above analysis results, we confirmed that TYROBP could be used to diagnose and predict MM prognosis, and verified that the high expression of TYROBP promoted the adhesion of MM cells and weakened their migration.

However, certain limitations should be pointed out in the present study. No analysis was made to compare TYROBP gene expression levels between metastatic and non-metastatic MM patient groups in the GSE data sets owing to the absence of metastatic and non-metastatic indicators. At the same time, there lacks of specific mechanisms and in vivo validation of the role of TYROBP overexpression in affecting the adhesion and migration of MM. We did, however, confirm that low TYROBP expression independently predicted poor prognosis of MM, and this was experimentally validated in clinical samples and in vitro studies. The mechanism by which TYROBP regulates cell adhesion affecting MM genesis and progression requires further experimental exploration, so as to shed novel lights and provide drug targets clinical treatment.

## Conclusion

Our study indicated that TYROBP was lowly expressed in MM, and its low expression predicted poor prognosis of MM patients. Multivariable COX regression revealed that TYROBP independently predicted the prognosis of MM. In addition, TYROBP was probably related to pathological development of MM via cancer-related signaling pathways, such as cell adhesion molecule signaling. TYROBP up-regulation promoted MM cell adhesion and decreased their migration. According to these results, TYROBP is a novel oncogene in MM, which may be a potential biomarker. However, further mechanism studies and in vivo experiments should be conducted for determining the clinical value of TYROBP.

### Supplementary Information

**Additional file 1:** **Table S1.** The characteristics of patients with multiple myeloma and healthy donors.

## Data Availability

Some data underlying this article are available in NCBI-GEO (http://www.ncbi.nlm.nih.gov/geo). The datasets were derived from sources in the public domain: GSE39754: https://www.ncbi.nlm.nih.gov/geo/query/acc.cgi?acc=GSE39754; GSE6477: https://www.ncbi.nlm.nih.gov/geo/query/acc.cgi?acc=GSE6477; GSE24080: https://www.ncbi.nlm.nih.gov/geo/query/acc.cgi?acc=GSE24080. Others are incorporated into the article and its online supplementary material. The data underlying this article are available in the article and in its online supplementary material.

## Abbreviations

MM: Multiple myeloma
GEO: Gene expression omnibus
DEGs: Differentially expressed genes
WGCNA: Weighted gene co-expression network analysis
GO: Gene Ontology
KEGG: Kyoto Encyclopedia of Genes and Genomes
PPI: Protein–protein interactions
ROC: Receiver operating characteristic
GSEA: Gene set enrichment analysis
RT-PCR: Reverse transcription-polymerase chain reaction
KM: Kaplan–Meier
NCCN: National Comprehensive Cancer Network
FBS: Fetal bovine serum
RIPA: Radio immunoprecipitation assay
PMSF: Phenylmethylsulfonyl fluoride
PVDF: Poly vinylidene fluoride
