# Supplementary material for: Low TYROBP expression predicts poor prognosis in multiple myeloma
Source: Cancer Cell Int. 2024 Mar 28;24:117. doi: 10.1186/s12935-024-03304-6 (PMC10979612; doi:10.1186/s12935-024-03304-6)
Supplement: Supplementary file 1 — Additional file 1: Table S1. The characteristics of patients with multiple myeloma and healthy donors. [file 12935_2024_3304_MOESM1_ESM.docx]

|  | Table S1 The characteristics of patients with multiple myeloma and healthy donors | | | | | | | | | |
| --- | --- | --- | --- | --- | --- | --- | --- | --- | --- | --- |
|  | Number | Age(year) | Gender | Serum β-MG(mg/L) | Serum albumin(g/L) | With extramedullary invasion or not | R-ISS stage | %Blasts(bone marrow) | Comorbidities | Cytogenetics/FISH |
|  | 1 | 58 | female | 3.19 | 41.99 | no | I | 19 | Hypertension | Normal |
|  | 2 | 63 | male | 31.68 | 26.47 | no | Ⅲ | 25 | None | Normal |
|  | 3 | 55 | male | 9.94 | 18.23 | no | Ⅲ | 14 | Hypertension | 1q21 amplification |
|  | 4 | 66 | male | 3.83 | 42.88 | no | Ⅱ | 10 | None | 1q21 amplification |
|  | 5 | 61 | male | 8.28 | 18.61 | no | Ⅲ | 30 | None | 1q21 amplification |
|  | 6 | 78 | male | 9.72 | 33.2 | yes | Ⅲ | 75 | None | 1q21 amplification |
|  | 7 | 69 | male | 2.45 | 36.4 | no | Ⅰ | 42 | None | 13q14 |
|  | 8 | 50 | female | 3.51 | 34.7 | yes | Ⅱ | 12 | Hypertension | Normal |
|  | 9 | 58 | male | 12.45 | 31 | yes | Ⅲ | 60 | Diabetes | 1q21 amplification |
|  | 10 | 58 | male | 19.48 | 46.6 | yes | Ⅲ | 54 | None | Normal |
|  | 11 | 82 | male | 15.68 | 37.85 | no | Ⅲ | 23 | None | Normal |
|  | 12 | 68 | female | 5.47 | 26.8 | no | Ⅲ | 28 | Hypertension | Normal |
|  | 13 | 63 | male | 8.81 | 31.14 | no | Ⅲ | 34 | None | Normal |
|  | 14 | 68 | male | 14.3 | 33.7 | no | Ⅲ | 67 | None | 13q14 |
|  | 15 | 49 | male | 2.47 | 44.89 | yes | Ⅰ | 54 | None | Normal |
|  | 16 | 46 | male | 8.51 | 34.8 | no | Ⅲ | 32 | None | 14q32 |
|  | 17 | 77 | male | 20.04 | 29.33 | no | Ⅲ | 47 | None | 1q21 amplification |
|  | 18 | 67 | female | 9.51 | 42 | yes | Ⅲ | 88 | Hypertension | 1q21 amplification |
|  | 19 | 53 | female | 18.17 | 43.87 | no | Ⅲ | 62 | None | 1q21 amplification |
|  | 20 | 55 | female | 5.35 | 28.81 | no | Ⅱ | 78 | None | 1q21 amplification |
|  | 21 | 57 | female | 11.58 | 30.08 | no | Ⅲ | 24 | None | Normal |
|  | 22 | 65 | male | 5.96 | 45.72 | no | Ⅲ | 24 | None | Normal |
|  | 23 | 40 | male | 1.1 | 41.7 | no | Ⅰ | 18 | None | Normal |
|  | 24 | 80 | male | 9.42 | 34.09 | no | Ⅲ | 60 | Chronic kidney failure | Normal |
|  | 25 | 70 | male | 108.38 | 23.74 | no | Ⅲ | 34 | None | Normal |
|  | 26 | 52 | female | 2.2 | 44.1 | no | Ⅱ | 90 | Diabetes | 1q21 amplification |
|  | 27 | 39 | male | 2.8 | 30.9 | no | Ⅱ | 13 | None | 1q21 amplification |
|  | 28 | 68 | male | 9.56 | 36.96 | yes | Ⅲ | 21 | Hypertension | Normal |
|  | 29 | 70 | female | 28.3 | 41.05 | no | Ⅲ | 23 | Hypertension | 1q21 amplification |
|  | 30 | 67 | male | 16.1 | 41.4 | no | Ⅲ | 67 | None | Normal |
|  | 31 | 74 | male | 18.85 | 35.79 | no | Ⅲ | 62 | None | Normal |
|  | 32 | 56 | female | 5.09 | 22 | no | Ⅱ | 34 | Renal calculi | Normal |
|  | 33 | 82 | male | 3.37 | 38.53 | no | Ⅰ | 31 | None | Normal |
|  | 34 | 79 | male | 6.98 | 33.73 | no | Ⅲ | 10 | None | 1q21 amplification |
|  | 35 | 55 | male | 5.94 | 35.71 | no | Ⅲ | 18 | Pulmonary nodules | Normal |
|  | 36 | 23 | male | - | - | - | - | - | - | - |
|  | 37 | 37 | male | - | - | - | - | - | - | - |
|  | 38 | 21 | female | - | - | - | - | - | - | - |
|  | 39 | 22 | male | - | - | - | - | - | - | - |
|  | 40 | 16 | male | - | - | - | - | - | - | - |
|  | 41 | 22 | male | - | - | - | - | - | - | - |
|  | 42 | 12 | male | - | - | - | - | - | - | - |
|  | 43 | 33 | male | - | - | - | - | - | - | - |
|  | 44 | 53 | male | - | - | - | - | - | - | - |
|  | 45 | 48 | male | - | - | - | - | - | - | - |
|  | 46 | 32 | female | - | - | - | - | - | - | - |
|  | 47 | 25 | male | - | - | - | - | - | - | - |
|  | 48 | 28 | female | - | - | - | - | - | - | - |
|  | 49 | 19 | male | - | - | - | - | - | - | - |
|  | 50 | 24 | female | - | - | - | - | - | - | - |
|  | 51 | 20 | male | - | - | - | - | - | - | - |
|  | 52 | 27 | male | - | - | - | - | - | - | - |
|  | 53 | 23 | female | - | - | - | - | - | - | - |
|  | 54 | 32 | male | - | - | - | - | - | - | - |
|  | 55 | 28 | male | - | - | - | - | - | - | - |
|  | 56 | 19 | male | - | - | - | - | - | - | - |
|  | 57 | 30 | male | - | - | - | - | - | - | - |
